# Osteoblast Response to Widely Ranged Texturing Conditions Obtained through High Power Laser Beams on Ti Surfaces

**DOI:** 10.3390/jfb15100303

**Published:** 2024-10-12

**Authors:** Federico Alessandro Ruffinatti, Tullio Genova, Ilaria Roato, Martina Perin, Giorgia Chinigò, Riccardo Pedraza, Olivio Della Bella, Francesca Motta, Elisa Aimo Boot, Domenico D’Angelo, Giorgio Gatti, Giorgia Scarpellino, Luca Munaron, Federico Mussano

**Affiliations:** 1 Department of Life Sciences and Systems Biology, University of Torino, Via Accademia Albertina 13, 10123 Torino, Italy; tullio.genova@unito.it (T.G.); martina.perin@unito.it (M.P.); giorgia.chinigo@unito.it (G.C.); luca.munaron@unito.it (L.M.); 2 Bone and Dental Bioengineering Laboratory, CIR Dental School, Department of Surgical Sciences, University of Torino, Via Nizza 230, 10126 Torino, Italy; ilaria.roato@unito.it (I.R.); riccardo.pedraza@unito.it (R.P.); federico.mussano@unito.it (F.M.); 3 Department of Mechanical and Aerospace Engineering, Politecnico di Torino, Corso Duca degli Abruzzi 24, 10129 Torino, Italy; 4 Biomec s.r.l. Colico, Via Nazionale Nord, 21/A, 23823 Colico, Italy; odellabella@biomec.net (O.D.B.); fmotta@biomec.net (F.M.); 5 Environment Park S.p.A. Plasma Nano-Tech, Via Livorno 60, 10144 Torino, Italy; elisa.aimoboot@envipark.com (E.A.B.); domenico.dangelo@envipark.com (D.D.); 6 Department of Science and Technological Innovation, University of Eastern Piedmont A. Avogadro, Viale Michel 11, 15121 Alessandria, Italy; giorgio.gatti@uniupo.it; 7 Department of Biology and Biotechnology “L. Spallanzani”, University of Pavia, Via Adolfo Ferrata 9, 27100 Pavia, Italy; giorgia.scarpellino@unipv.it

**Keywords:** titanium implants, surface roughness, cell adhesion, protein adsorption, surface free energy

## Abstract

Titanium and titanium alloys are the prevailing dental implant materials owing to their favorable mechanical properties and biocompatibility, but how roughness dictates the biological response is still a matter of debate. In this study, laser texturing was used to generate eight paradigmatic roughened surfaces, with the aim of studying the early biological response elicited on MC3T3-E1 pre-osteoblasts. Prior to cell tests, the samples underwent SEM analysis, optical profilometry, protein adsorption assay, and optical contact angle measurement with water and diiodomethane to determine surface free energy. While all the specimens proved to be biocompatible, supporting similar cell viability at 1, 2, and 3 days, surface roughness could impact significantly on cell adhesion. Factorial analysis and linear regression showed, in a robust and unprecedented way, that an isotropic distribution of deep and closely spaced valleys provides the best condition for cell adhesion, to which both protein adsorption and surface free energy were highly correlated. Overall, here the authors provide, for the first time, a thorough investigation of the relationship between roughness parameters and osteoblast adhesion that may be applied to design and produce new tailored interfaces for implant materials.

^‡^ These authors contributed equally to this work.

## 1. Introduction

The high technical knowledge achieved [1] has made dental implants the prevailing therapeutic option for replacing missing teeth. Titanium (Ti) is used in the majority of commercially available implants, acting as the gold standard material [2,3], which benefits from a low elastic modulus, good corrosion resistance, and a relatively low cost compared to other metallic alloys [4]. The market is dominated by a series of Ti-based bulk materials ranging from commercially pure (cp) Ti to more mechanically resistant alloys such as titanium–aluminum–vanadium (Ti6Al4V) [5] and even titanium–zirconium [6]. Independently of their composition, however, the biological interface of all these Ti-based materials is represented by the inherent thin oxide layer that, originated from the high affinity of Ti for oxygen, reduces corrosion and remarkably mimics the ceramic nature of bone [7], which appears to explain also the excellent biocompatibility of massive ceramic implants (based on zirconia oxide) [8].

In the last decades, to enhance osseointegration (i.e., the direct connection between bone and implant), major advancements have been attained by augmenting the surface roughness of the device [4], thus increasing bone-to-implant contact and improving clinical success rates [9,10]. Micro-rough implants resulted superior to smooth ones [11], proving that micrometric scale modifications act directly on the biological response.

Interestingly, the clinical outcome has been correlated in vitro to the early cell behavior of bone precursors [12]. In particular, surface roughness of titanium implants strongly increases cell adhesion, proliferation, and differentiation of osteogenic cells [13,14,15]. Hence, establishing which cues are fundamental in guiding cell behavior has become a paramount task, albeit an unmet one, when assessing possible novel implant surface candidates.

Unfortunately, difficulties arise whenever a comprehensive theoretical model is to be proposed. Surface topography, even in its essentially physical definition at the micrometer scale, cannot be separated from other descriptors such as wettability, which, in turn, contributes to the surface free energy (SFE) assessment. Systematic investigations of all these descriptors on a large range of different surface types have never been reported, and contradictory data are often available. For instance, the liquid–solid contact angle of clinically marketed implants ranges widely [16], despite their equal success rate.

Through melting and evaporation of an established amount of material from the surface, high-power laser beams are capable of generating a complex and well-defined surface geometry [17]. Moreover, by adding to the process a gas jet that removes the melted material [18], the surface remains without chemical residues, possibly causing unpredicted biological effects. Thus, specific patterns and controlled chemical interfaces may be prepared with a precision level never achieved with the traditional grit blast and acid etching techniques.

Given these premises, the authors wanted to test the effect surface texture has on cell adhesion as a recognized measure of early biological response. To this aim, among the numerous roughening strategies available [5], high-power laser beams [19] were selected to produce surfaces endowed with a series of pre-designed texture features that encompass an unprecedented range of roughness values, with both random and geometric patterns. The null hypothesis of the study was that cell adhesion did not vary for different values and combinations of the parameters used for surface laser patterning, and hence that such a biological response could not be predicted from the different roughness features of the titanium substrates.

## 2. Materials and Methods

### 2.1. Sample Preparation

Eight different surface modifications, each one in ten technical replicates, were obtained out of 80 grade 4 cp titanium disks (Biomec s.r.l. Colico, Como, Italy), through a high-power laser machine (LASER P 400, GF, Schaffhausen, Switzerland), whose process parameters are reported in Table 1.

**Figure 1:**
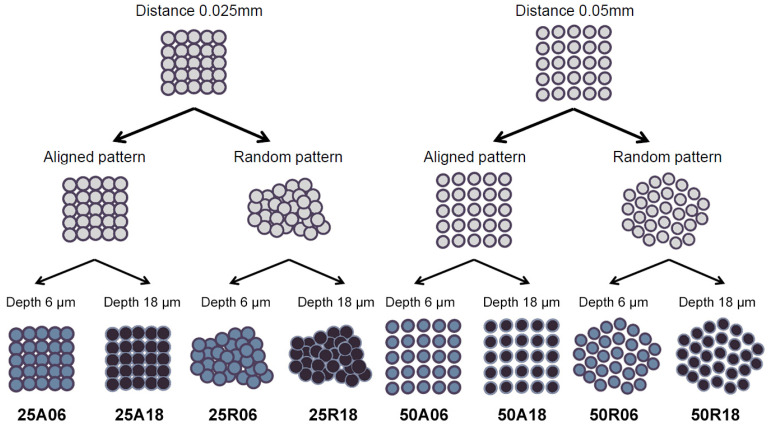
Scheme of the factorial experimental design followed for surface modification of titanium disks. Throughout this work, titanium samples are identified using an alphanumeric ID encoding these three features, in the following order: inter-pit distance (25 or 50 μm), pattern type (A for aligned or R for random), and pit depth (6 or 18 μm).

The laser beam was applied on the titanium surfaces to carve 50 μm diameter pits, whose relative distance, position, and depth were modulated to create the eight different textures. In particular, the sample disks were prepared following the three-factor experimental design depicted in Figure 1, in which two levels were set for each factor:

- the average distance between the pits (either 0.025 mm or 0.05 mm),
- the pattern type (either an aligned grid or a random pattern),
- the depth of the pits (either 6 μm or 18 μm).

All through this work, each surface modification has been assigned to a unique identifier consisting of the ordered values taken by these three independent variables during laser application: inter-pit distance in micrometers (either 25 or 50), pattern type (either A for aligned or R for random), and pit depth in micrometers (either 6 or 18). For instance, ID 25R06 refers to a titanium disk patterned with pits spaced 25 μm from each other (on average), randomly distributed on the surface, and 6 μm deep.

### 2.2. SEM Analysis

The surface morphology of the samples was investigated qualitatively through scanning electron microscopy (SEM) using a Phenom XL G2 Desktop SEM (Thermo Fisher Scientific, Milan, Italy). Before observation, the samples were cleaned in acetone (179124 Sigma), rinsed in MilliQ ultrafiltered water (Medica EDI, ELGA High Wycombe, UK), and finally dried. The detector was configured in full BSD mode, the working distance was fixed at 8.43 mm, the accelerating voltage was set at 15 kV, and the chamber vacuum environment was maintained at 10 Pa. For each disk, at least three photographs were taken at a fixed magnification (1000×).

### 2.3. Roughness Evaluation

The surface topography of each sample was quantitatively characterized by a 3D optical profilometer (Sensofar, Barcelona, Spain), according to ISO 25178 [20], which provides geometrical product specifications (GPS) for the analysis of 3D areal surface texture [21]. See Supplementary Materials Table S1 for a complete list and a brief description of the eleven roughness parameters used for the analysis.

### 2.4. Wettability and SFE

Titanium wettability was assessed by measuring the optical contact angle (OCA) through the sessile drop technique, employing an Attension Theta Lite optical tensiometer (Biolin Scientific Gothenburg, Sweden). Volumes of 1 μL of two different liquid probes—namely water and diiodomethane—were dropped over each titanium texture, and their images were acquired using the integrated camera. Drop profiles were then analyzed using dedicated software (OneAttension ver. 4.1.2) and OCA (θ) at the liquid–solid interface was determined. Each OCA measurement, for both water and diiodomethane, was repeated 16 times on different areas of the sample. Average OCA was then used to estimate the SFE ($\gamma_{s}$) of the titanium substrates according to the Owens–Wendt–Rabel–Kaelbel (OWRK) method [22]. For each titanium sample, the following equation was evaluated for both water and diiodomethane,

$$\frac{\gamma_{l}(1+\cos\theta )}{2\sqrt{\gamma_{l}^{D}}}=\sqrt{\gamma_{s}^{D}}+\sqrt{\gamma_{s}^{P}}\sqrt{\frac{\gamma_{l}^{P}}{\gamma_{l}^{D}}}$$

where θ is the average OCA, $\gamma_{l}=\gamma_{l}^{P}+\gamma_{l}^{D}$ is the liquid surface tension, and $\gamma_{s}=\gamma_{s}^{P}+\gamma_{s}^{D}$ is the solid SFE, both the latter divided into their polar (P) and dispersive (D) components. The previous equation allows calculation of the two SFE terms by simple linear regression, provided the properties of the liquids are known. In this work, the literature values reported in Table 2 were used [23]:

With highly hydrophobic surfaces, the OWRK method may return negative slopes for the fitted line. In those cases, a slope of zero is usually assumed ($\gamma_{s}^{P}=0$) and a horizontal line is fitted instead [22].

### 2.5. Protein Adsorption Assay

To assess protein adsorption, titanium disks were incubated with 2% fetal bovine serum (FBS) in PBS for 20 min. The samples were then washed with PBS and placed in a sterile 24-well cell culture plate. Finally, adsorbed proteins were quantified through the biuret test using bicinchoninic acid (BCA) for the colorimetric analysis. Specifically, the 24-well cell culture plate containing the reagents was incubated for 40 min at 37 °C, and then the optical density at 562 nm was measured [24]. Samples containing only the reagents without the analyte and samples of known concentration were used, respectively, as blank and standard samples for calibration.

### 2.6. Cell Culture

The MC3T3-E1 murine pre-osteoblastic cell line was cultured in DMEM (Dulbecco’s modified Eagle’s medium) supplemented with 10% fetal bovine serum (FBS), 100 U/mL penicillin, and 100 μg/mL streptomycin under a humidified atmosphere with 5% CO_2_ at 37 °C. Cells were grown in Petri dishes and passaged at sub-confluency to prevent contact inhibition. Cells were detached with trypsin, washed with phosphate-buffered saline (PBS), and suspended in DMEM as previously described [25].

### 2.7. Cell Adhesion Assay

Cell adhesion on titanium samples was assessed using a 24-well plate as support. Cells were detached with trypsin for 3 min and seeded at a density of 2500 cells/disk in 50 μL of growth medium. The 24-well plate was kept at 37 °C, 5% CO_2_ for 15 min. After 15 min, non-adherent MC3T3-E1 cells were removed by rinsing twice in PBS. Cells were fixed in 4% paraformaldehyde for 10 min and then washed with PBS. Subsequently, cells were stained with DAPI (4′, 6-diamidino-2-phenylindole) 1 μM (Molecular Probes, Eugene, CA, USA) for 15 min to visualize cell nuclei. Cell adhesion assays were performed in biological triplicates (n=3 independent experiments for each surface type). Nuclei of cells stained with DAPI were counted using fluorescence microscopy as a measure of cell adhesion on titanium disks, as previously reported [26,27].

### 2.8. Cell Viability Assay

Cells were cultured at 37 °C in a humidified environment with 5% CO_2_ for 24 and 72 h. ATP release, indicative of cell viability, was measured using the Cell Titer GLO assay (Promega, Madison, WI, USA), as described previously [28].

### 2.9. Statistical Analysis

Three-way ANOVA was used to analyze the influence on cell adhesion of the three parameters controlled during titanium disk manufacturing and laser surface modification (i.e., inter-pit distance, pattern type, and pit depth). Multiple linear regression (MLR) was used to derive a mathematical model predicting cell adhesion from the substrate roughness data. To remove multicollinearity from the dataset, the variance inflation factor (VIF) was used as a suitable criterion. For both ANOVA and MLR, type III (aka partial) sums of squares were used for variability estimation and adjusted *p*-value computation. Pearson and Spearman correlation coefficients were used to analyze the correlation between cell adhesion and protein adsorption or SFE. Mean and standard error of the mean (SE) were used to provide estimates for cell adhesion, viability, protein adsorption levels, OCA, and SFE. All statistical calculations were performed in the RStudio environment by means of custom R scripts.

## 3. Results

### 3.1. Surface Characterization of the Samples

**Figure 2:**
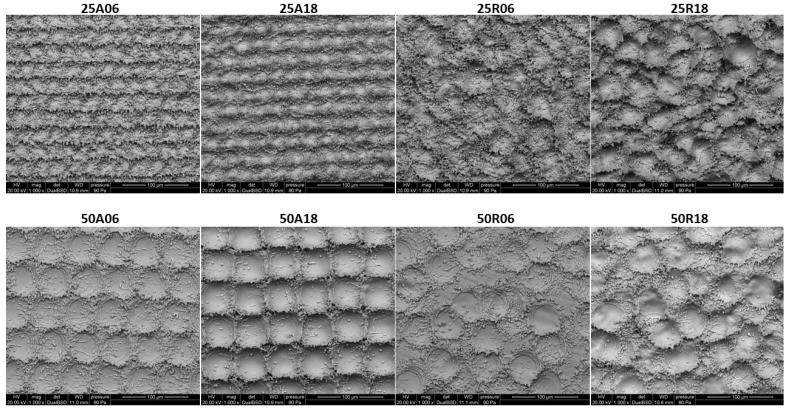
SEM images showing the surface topography of the titanium samples at high magnification (1000×). Each surface is named after the alphanumeric ID defined above, which consists of the values of pit spacing (25 or 50 μm), pattern type (Aligned or Random), and pit depth (6 or 18 μm), respectively.

The eight different surface textures obtained on Ti disks underwent SEM analysis to qualitatively assess their topography (Figure 2), which confirmed the correct implementation of the conceptual design resulting from the factorial combination of pit spacing, pattern type, and pit depth features (Figure 1).

The specimens were then quantitatively characterized in terms of roughness according to eleven descriptors following the ISO 25178 standard for 3D surface texture specification and measurement (for a description of the roughness parameters, refer to Table S1). The results thereof are shown in Table 3.

As for SFE, regardless of the particular laser processing, all the substrates showed a pronounced hydrophobicity, such that the polar component of the surface energy was negligible compared to the dispersive one. For this reason, $\gamma_{s}$ values reported in Table 4 correspond to the dispersive component only ($\gamma_{s}\approx \gamma_{s}^{D}$). Despite the substantial absence of a polar component, laser processing allowed a considerable modulation of the SFE values acting on the dispersive component alone.

### 3.2. Early Biological Response

**Figure 3:**
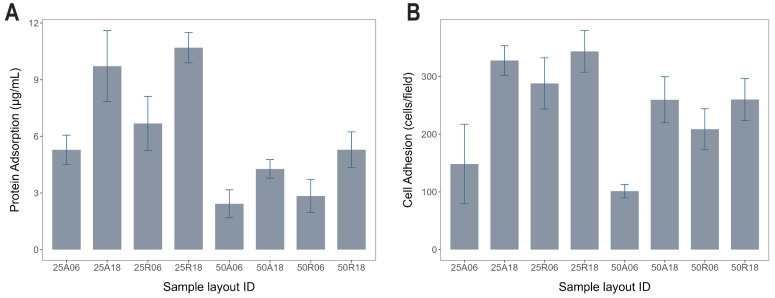
Protein adsorption and cell adhesion measures. (**A**) Data from three independent protein adsorption assays are represented as mean ± SE of the amount of adsorbed protein per volume (µg/mL), for each different titanium surface. (**B**) Data from three independent cell adhesion assays are represented as mean ± SE of the number of counted cells per field of view, for each different titanium surface.

Protein adsorption was evaluated on the eight surface modifications. Specifically, adsorption data of the fetal bovine serum proteins on the titanium samples are reported in Figure 3A. Cell adhesion was estimated by manually counting the number of adherent MC3T3-E1 cells stained with DAPI. Results from each experiment (three replicates) are reported in Table 5 and shown in Figure 3B as a bar chart.

### 3.3. Cell Viability

**Figure 4:**
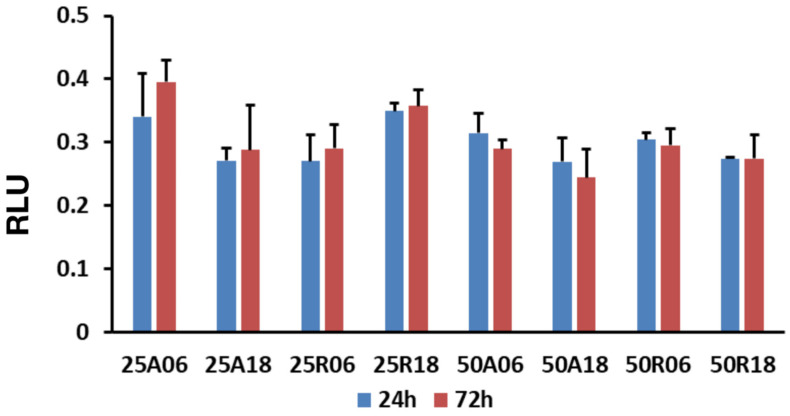
Cell viability at 24 h and 72 h. Data from three independent experiments are presented as mean ± SE for each different titanium surface (RLU = relative light units).

All the surfaces supported the growth of MC3T3-E1, showing no toxicity. No statistically significant differences were found according to ANOVA at both the time points, as portrayed in Figure 4.

### 3.4. Experimental Design-Based Factorial Analysis

**Figure 5:**
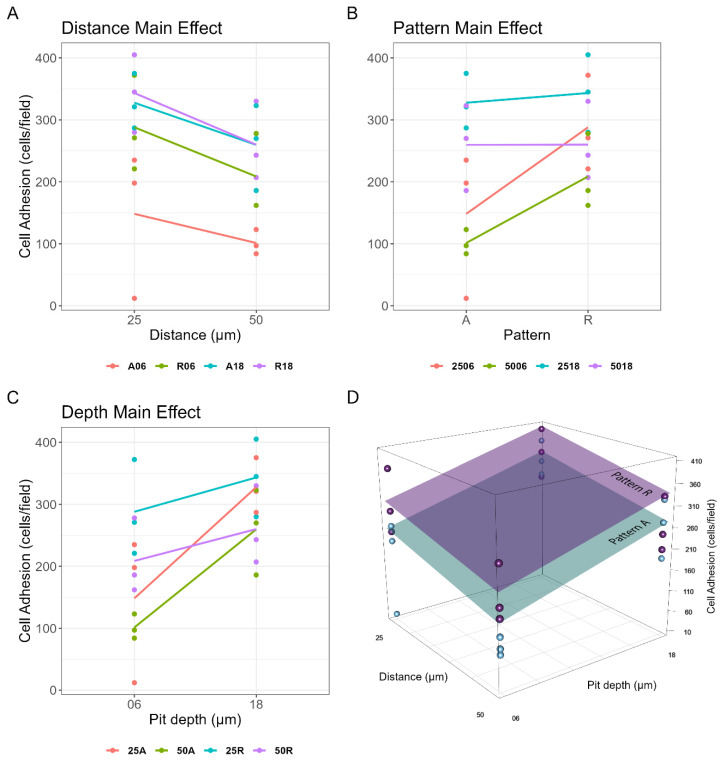
Factorial analysis. Main effects on cell adhesion of: (**A**) inter-pit distance (*p*-value = 2.55 × 10^−2^), (**B**) pattern type (*p*-value = 3.32 × 10^−2^), and (**C**) pit depth (*p*-value = 9.67 × 10^−4^). (**D**) A 3D representation of the global regression model. Light-blue and violet planes represent the linear model equation evaluated for aligned and random pattern, respectively.

A three-way ANOVA was used to study the effects on cell adhesion of the three parameters set during the laser texturing of the titanium surfaces. The analysis showed no statistically significant interactions among factors, except for the interaction term between pit depth and pattern type, whose *p*-value resulted very close to the conventional 5% threshold (*p*-value = 0.052). For this reason, a plain additive model was used to study the main effects of the three independent variables, showing that all of them significantly affected cell adhesion, although to varying degrees (Table 6 and Figure 5). More details about the analysis and the related statistics can be found in Supplementary Materials (Section S2).

### 3.5. Multiple Linear Regression Based on Roughness Parameters

**Figure 6:**
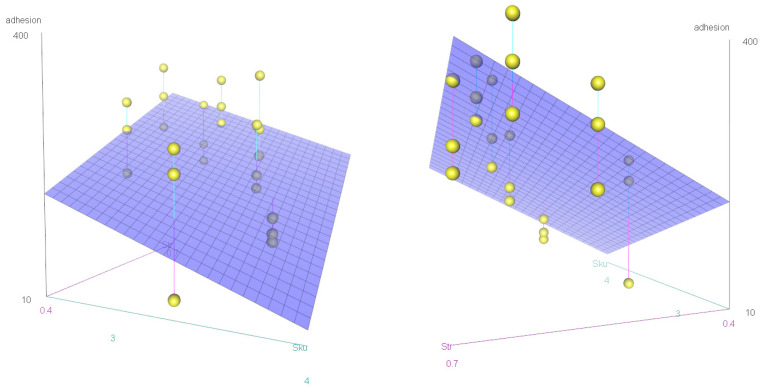
Graphical representation of the plane model. Two different views of the same plane given by Equation (1) linking cell adhesion data points (yellow dots) to the surface roughness properties of the titanium disks. Multiple linear regression analysis returned only two non-redundant and statistically significant coefficients out of the eleven initial roughness descriptors ($\beta_{Str}$ coefficient: *p*-value = 7.10 × 10^−3^; $\beta_{Sku}$ coefficient: *p*-value = 3.57 × 10^−2^.

To validate and further extend our previous results, we asked if MC3T3-E1 cell adhesion on Ti disks could also be predicted starting from a general quantification of their surface roughness, even in the absence of any a priori information about the manufacturing process. Out of the eleven ISO parameters evaluated from the 3D optical rendering of the sample surfaces (Table 3), only three—Sku, Ssk, and Str—were retained as mutually non-redundant regressors (more details on this model selection procedure can be found in Supplementary Materials, Section S3). However, when used as independent variables in multiple linear regression (MLR) analysis, only Str (an index of surface isotropy) and Sku (the kurtosis of height distribution) were able to explain a relevant and statistically significant amount of variance in the original dataset (Table S3). These two parameters were then used for the definition of a mathematical model suitable to predict cell adhesion (Equation (1)), the graphical representation of which is shown in Figure 6.

(1) adhesion=149.77+647.97⋅Str−95.02⋅Sku

### 3.6. Correlation between Cell Adhesion, Protein Adsorption, and SFE

**Figure 7:**
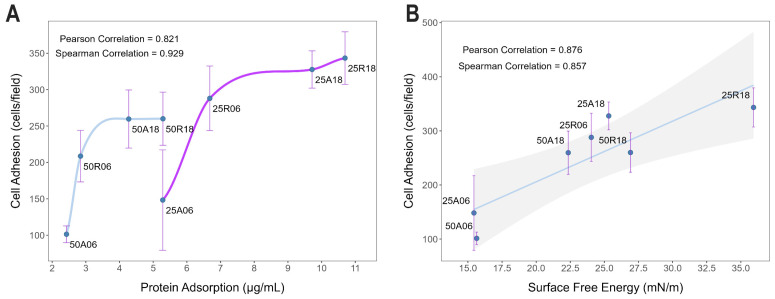
Correlation analysis between cell adhesion, protein adsorption, and SFE. (**A**) Scatterplot and correlation analysis between protein adsorption and cell adhesion data. The numerical values of the correlation coefficients shown in the graph refer to the overall correlation analysis between the two datasets (Pearson correlation coefficient $\rho_{P}=0.82$, p-value=0.012; Spearman correlation coefficient $\rho_{S}=0.93$, p-value=0.002), while two independent loess curves were used to highlight the nearly deterministic relationship between protein adsorption and cell adhesion within the single subset of surfaces with 50 μm inter-pit distance (cyan curve) and 25 μm spacing (magenta curve). (**B**) Scatterplot and correlation analysis between SFE and cell adhesion data. The best fitting line (in blue) and the 95% confidence interval (shaded in gray) are superimposed on the data points (Pearson correlation coefficient $\rho_{P}=0.88$, p-value=0.009; Spearman correlation coefficient $\rho_{S}=0.86$, p-value=0.024).

Cell adhesion and protein adsorption charts share remarkable resemblance (Figure 3). Indeed, correlation analysis between cell adhesion and protein adsorption data confirmed their high degree of overall association, with a Pearson correlation coefficient $\rho_{P}=0.82$ (p-value=0.012) and a Spearman correlation coefficient $\rho_{S}=0.93$ (p-value=0.002). Correlation values were even larger if the two batches of substrates with 25 μm and 50 μm of inter-pit distance were analyzed separately, resulting in almost unitary Spearman correlation coefficients, as shown in Figure 7A. Likewise, correlation analysis showed how SFE is also an excellent statistical predictor of cell adhesion for these materials (Pearson correlation coefficient $\rho_{P}=0.88$, p-value=0.009).

## 4. Discussion

In dental implantology, the role of titanium surface features on early cell response has been investigated extensively in the last few decades, highlighting particularly the importance of roughness and wettability [29,30]. While “roughened” surfaces are known to enhance osseointegration, standard surface parameters have not been achieved so far, making the very definition of surface roughness somehow unclear. Quite appropriately, Matteson et al. [31] noted that “to gain a more holistic understanding of complex surface texture it can be useful to remove gross form, then separate and independently quantify the waviness (large scale) and roughness (fine scale) vertical components of surface topography via digital filtering”. In addition, with the exception of the most recent papers [32,33], there is a marked prevalence in literature regarding simple roughness parameters (Sa or Ra) that reduce the information available to a single value, even if a complex hierarchical structure should be present. This becomes a serious pitfall when comparing data across multiple studies, generating inconsistencies and hindering knowledge.

This work aims at presenting a more comprehensive approach for studying the surface texture correlated with cell adhesion. MC3T3-E1 cells were adopted following a well-established in vitro model of the early biological response elicited by intraosseous fixtures [34,35]. Since surface roughening is usually achieved through acid etching combined with sand blasting [3,36], which could not allow a precise control of the texture pattern, here, a high-intensity laser was used to etch pits into the titanium substrate. This technique allowed the formation of a Ti oxide coating on all the samples, as assessed through energy dispersive X-ray analysis (see Section S5 of the Supplementary Materials). A factorial scheme was implemented, in which three independent parameters—namely inter-pit distance, pattern type, and pit depth—were varied, each on two discrete levels, for a total of eight different textures. Notably, the surfaces obtained were characterized by an unprecedented broad range of Sa and Sz values, with the purpose of exploring conditions that are seldom found in the literature. Taking advantage of the a priori information on sample preparation, a three-way ANOVA was carried out to investigate the effects on cell adhesion of the three parameters controlled during laser-texturing. This allowed the rejection of the null hypothesis that different laser processing of titanium surfaces did not influence the cellular response. In particular, pit depth proved to be the most influential factor for cell adhesion ($\beta_{depth}=111.08$, $p\text{-}\mathrm{value}=9.67\times 10^{-4}$). Specifically, deep pits allowed for a more effective cell adhesion, especially when combined with an aligned pattern (Figure 5C). On the other hand, moving from an aligned pattern to a random one significantly improved cell adhesion only in the presence of shallow pits (Figure 5B), thus confirming the interaction between pit depth and pattern type. In other words, a random pit distribution can compensate (at least partially) for their reduced depth, and, vice versa, increasing pit depth is sufficient to improve cell adhesion and make the influence of the pattern negligible. Lastly, reducing the average pit spacing further increased the number of adherent cells, regardless of (i.e., do not interact with) any other factors (Figure 5A, $\beta_{distance}=-69.42$, $p\text{-}\mathrm{value}=2.55\times 10^{-2}$). Accordingly, the best cell adhesion performances were given by titanium disks with 0.025 mm of average inter-pit distance, 18 μm of pit depth, and a random pattern—even though this last factor seems to have poor or no influence on cell adhesion when pits are that deep (see blue line in Figure 5B). Specifically, 25R18 featured an average count of adherent cells of 343.33 ± 36.09 cells/well, thus yielding an overall improvement of nearly 3.5 times if compared to the worst scenario in 50A06, which provided just 101.33 ± 11.46 adherent cells/field on average. It is worth noting that, while this ratio could already be inferred from cell adhesion measurements alone (Figure 3B), the three-way ANOVA provided the underlying mathematical model that allowed the specific contribution of each factor to be dissected.

Since noncontact areal methods may ameliorate the estimation of surface texture [37], each of the eight surface modifications underwent roughness analysis according to eleven roughness parameters (ISO 25178) through optical profilometry. MLR was used to find the most effective and statistically significant cell adhesion predictors among these surface texture descriptors. Since multicollinearity (i.e., the correlation among regressors) needs to be kept as low as possible to properly accomplish such an analysis, we removed the most redundant descriptors based on VIF estimation and only two mutually uncorrelated regressors with a significant influence on cell adhesion—Str and Sku—took part in the final mathematical model, thus allowing us to also reject the study’s second null hypothesis that cellular response could not be predicted from surface roughness features. The emergence of these two descriptors is in agreement, although attained in a different way, with a previous report [31] stating that “the implementation of spacing parameters, which assess the lateral or horizontal characteristics of a surface can provide valuable insight into the overall surface texture”.

More specifically, Str, also called the texture aspect ratio of the surface, is a measure of the spatial directionality of the surface texture whose values range between 0 and 1, in the presence of a dominant lay or for a spatially isotropic texture, respectively. As for the titanium samples used in this study, Str effectively discriminated the four aligned patterns from the four random configurations ($Str_{A}=0.553\pm 0.044$ vs $Str_{R}=0.643\pm 0.019$, from Table 3). Consistent with the initial factorial analysis for which a random arrangement of pits was a significant factor for the increase in cell adhesion, Equation (1) predicts that cell adhesion is likely to be improved by surfaces featuring higher Str values.

Sku is instead the *kurtosis of height distribution*. Compared to a normal distribution of height values along the z-axis, leptokurtic (Sku>3) distributions are associated with an excess of outliers relative to the mean plane (too many or too high/deep defects), while platykurtic (Sku<3) distributions are more concentrated around the mean, producing fewer and/or less extreme outliers. Unexpectedly, applied to our sample set (Table 3), Sku classified the four surfaces with shallow pits as leptokurtic ($Sku_{06}=3.439\pm 0.084$) and the four surfaces with deep pits as platykurtic ($Sku_{18}=2.772\pm 0.119$). However, as the coefficient of Sku is negative in Equation (1), platykurtic distributions of height values (i.e., deep pits) were the ones that provided the best cell adhesion, again showing a substantial agreement between the a priori categorical model (three-way ANOVA) and the a posteriori continuous one (MLR).

The reason why deeper pits were associated with height distributions with a negative excess of kurtosis is less obvious but can be better understood when considering the actual effects of laser modification of the titanium surface. Indeed, whenever a pit is created, part of the removed material rearranges to form peaked protrusions surrounding the edges of the pit (Figure S2). These positive deviations from the mean plane are the main determinants of the generally positive skewness values that characterize all the surfaces from our sample set (average Ssk=0.426±0.078) and they are more likely to give rise to statistical outliers—and therefore to a large kurtosis—when the valleys are shallow. Besides the presence of the pits, we must consider that even these “edge peaks” could have an important role in cell adhesion, and, while in the a priori analysis this aspect was somehow incorporated into the “pit depth” factor, they may represent the reason why in the a posteriori analysis Sku was a more explicative descriptor than the mere estimation of valley depth (Sv) or peak height (Sp).

Taken together, the two statistical analyses confirmed that higher levels of roughness (i.e., high densities of deep and randomly distributed pits) provide the titanium surface with better performance in terms of MC3T3-E1 cell adhesion, while also quantifying the impact of each surface feature on the biocompatibility of lased titanium substrates.

To gain a first insight into the cellular mechanisms underlying the different cell adhesion profiles exhibited by the eight surface textures in this study, protein adsorption assays were also performed over the same samples. The initial response when blood encounters an artificial surface is the adsorption of plasma proteins [22,38], thus representing a fundamental player of the complex healing process that occurs at the interface between a biomaterial and the living recipient [39]. Protein adsorption leads very rapidly to the formation of a protein layer that is crucial for cells to recognize the implant as self [40]. For this reason, protein coating is one of the most important determinants of implant material biocompatibility, especially in the context of implant functionalization and new medical device development [41,42]. Protein adsorption is influenced by many different factors, such as surface features (roughness, wettability, charge exposure), protein properties (surface charge, hydrophilicity, structure), and also solution parameters (composition, pH, temperature) [41,43,44]. For example, substrate wettability is a major driving force for adsorption [45,46]. Titanium is known for its affinity for proteins [47]. In particular, the roughness of titanium substrates affects adsorption [48]. Surface features like protuberances and peaks are topographical characteristics that can influence protein adsorption, among others. Actually, also surface pores in the meso- and nano-range should be taken into consideration for surface roughness [49]. Here, protein adsorption was shown to be an excellent statistical predictor of cell adhesion, which also correlated with the SFE, in accordance with previous reports [50,51]. Interestingly, correlation values were even larger if the two batches of substrates with 25 μm and 50 μm of inter-pit distance were analyzed separately, resulting in almost unitary Spearman correlation coefficients, as shown in Figure 7A, where the magenta curve (25 μm batch) closely mirrors the trend of the cyan one (50 μm batch), but shifted towards higher protein adsorption and cell adhesion values. The influence of pit density is reasonable if considering that, for a given material, surface development—intended as the surface area contributed by the texture as compared to the planar area—is usually a major determinant factor of protein adsorption properties by a substrate. Notably, the fact that Spearman correlation coefficients were systematically larger than Pearson’s suggests a nonlinear relationship between these two biocompatibility indexes.

## 5. Conclusions

This paper provides an innovative insight into the very debated topic regarding the influences of different surface parameters on the biological response. The roughness of the surfaces tested was unprecedented, as it ranged from Sa=3.2 μm to Sa=26.2 μm, which is far beyond the recommendation of the optimal roughness accepted in dental implantology [52]. Thus, the experimental design adopted—combined with a powerful and systematic statistical analysis—allowed the alternative hypotheses underlying the study to be accepted, whereby (a) the particular laser processing of titanium surfaces can significantly influence cell adhesion, (b) it is possible to predict this cellular response from surface roughness measurements (particularly from Sku and Str descriptors), (c) there is a correlation between cell adhesion and protein adsorption, and (d) between cell adhesion and surface free energy. This may contribute to the progress of the theoretical knowledge in the field, in which the prevailing parameters adopted are Sa (or Ra) and Sdr. As a limitation of the study, on the other hand, it is worth noting that no real application of too rough surfaces can occur due to the likely release of debris during the implant placement in the recipient bone. Future perspectives entail the customization of the surface texture according to the cell type. Ideally, the interface of an artificial biomaterial should mimic the extracellular matrix as much as possible to achieve the best integration. Hence, within the limits of the industrial feasibility, it is conceivable that dental implants will be endowed with different intraosseous and transmucosal surfaces optimized for enhancing the adhesion—respectively—of the osteoblasts (bone) and the fibroblasts/epithelial cells.

## Figures and Tables

**Table 1:** **Process parameters.** Specifications of the manufacturing process for surface modification of titanium samples used in the study.

Surface ID	Power	Frequency (kHz)	Application Time (s)	Interpoint Distance (mm)	Depth (µm)	Pattern (Aligned/Random)
**1**—**25A06**	100%	130	28	0.025	6	A
**2**—**25A18**	100%	130	38	0.025	18	A
**3**—**25R06**	100%	130	28	0.025	6	R
**4**—**25R18**	100%	130	38	0.025	18	R
**5**—**50A06**	100%	130	9	0.050	6	A
**6**—**50A18**	100%	130	11	0.050	18	A
**7**—**50R06**	100%	130	9	0.050	6	R
**8**—**50R18**	100%	130	11	0.050	18	R

**Table 2:** Liquid surface tension values used for SFE computation. For both liquids, the total surface tension $\gamma_{l}$ is the sum of the polar ($\gamma_{s}^{P}$) and dispersive components ($\gamma_{s}^{D}$).

Liquid	$\gamma_{l}^{P}$ (mN/m)	$\gamma_{l}^{D}$ (mN/m)	$\gamma_{l}$ (mN/m)
**Water**	43.70	29.10	72.80
**Diiodomethane**	2.60	47.40	50.00

**Table 3:** Roughness measures. Values of the eleven ISO 25178 descriptors used to characterize the eight different titanium surfaces. Sa, Sp, Sq, Sv, Sz, and Sal are given in μm; Str, Sdq, Ssk, and Sku are dimensionless pure numbers; Sdr is expressed as percentage (%).

Surface	*Sa*	*Sku*	*Sp*	*Sq*	*Ssk*	*Sv*	*Sz*	*Sdq*	*Sdr*	*Sal*	*Str*
**25A06**	4.491	3.234	35.79	5.652	0.274	28.51	64.30	2.578	225.70	6.841	0.447
**25A18**	17.530	3.071	80.41	22.210	0.063	81.43	161.80	5.688	907.20	23.900	0.662
**25R06**	5.196	3.426	36.75	6.547	0.480	39.85	76.60	2.468	207.00	12.310	0.629
**25R18**	26.210	2.617	99.87	32.300	0.247	123.80	223.70	7.456	1497.00	26.140	0.644
**50A06**	3.407	3.647	68.02	4.037	0.651	28.92	96.94	1.343	68.16	11.030	0.542
**50A18**	8.866	2.547	104.80	10.510	0.694	68.15	173.00	2.824	251.40	12.620	0.562
**50R06**	3.288	3.447	56.38	3.961	0.410	29.56	85.93	1.350	65.61	12.620	0.604
**50R18**	11.140	2.853	102.40	13.380	0.587	79.93	182.30	3.329	331.80	18.410	0.693

**Table 4:** SFE of the different titanium textures, as evaluated using the OWRK method. For each substrate, OCA was measured for water and diiodomethane. Because of the hydrophobic nature of the titanium surfaces, the values reported here are attributable to the dispersive component only.

Surface	$\gamma_{s}$ (mN/m)	SE (mN/m)
**25A06**	15.44	0.45
**25A18**	25.32	1.23
**25R06**	24.04	0.57
**25R18**	35.93	1.81
**50A06**	15.64	0.01
**50A18**	22.35	0.99
**50R18**	26.91	1.28

**Table 5:** Cell adhesion dataset. Values were obtained by direct cell counting of adherent cells per field of view (cells/field) for each tested titanium disk.

Surface	25A06	25A18	25R06	25R18	50A06	50A18	50R06	50R18
**Replicate 1**	198	321	372	280	97	270	186	243
**Replicate 2**	12	287	271	405	123	186	162	330
**Replicate 3**	235	375	221	345	84	323	278	207

**Table 6:** Summary statistics of the general linear model used to fit cell adhesion data. Since no significant interactions emerged from preliminary analyses, a purely additive model was used, namely: adhesion ~ distance+pattern+depth.

Coefficients	Estimate	Std. Error	*t*-Value	*p*-Value
**distance**	−69.42	28.75	−2.414	2.55 × 10^−2^
**pattern**	65.75	28.75	2.287	3.32 × 10^−2^
**depth**	111.08	28.75	3.864	9.67 × 10^−4^

## Data Availability

The original contributions presented in the study are included in the article/supplementary material, further inquiries can be directed to the corresponding author.

## Supplementary Materials

The following supporting information can be downloaded at: https://www.mdpi.com/article/10.3390/jfb15100303/s1, Table S1: ISO 25178 parameters; Table S2: statistics for the general linear model used to fit the data; Table S3: MLR summary statistics; Table S4: carbon relative presence; Table S5: oxygen relative presence; Table S6: titanium relative presence; Figure S1: correlation among regressors; Figure S2: edge peaks; Figure S3: elemental analysis of titanium disc surfaces.
