# Supplementary material for: Osteoblast Response to Widely Ranged Texturing Conditions Obtained through High Power Laser Beams on Ti Surfaces
Source: J Funct Biomater. 2024 Oct 12;15(10):303. doi: 10.3390/jfb15100303 (PMC11508658; doi:10.3390/jfb15100303)
Supplement: Supplementary file 1 [file jfb-15-00303-s001.zip › jfb-3145696-supplementary.pdf]

Journal of
Functional Biomaterials

MDPI

1. ISO 25178 parameters

Table S1. ISO 25178 parameters. Type, symbol, and description of the ISO 25178 parameters used in this study, according to the standards of Geometrical Product Specifications (GPS) for the analysis of 3D areal surface textures.

Parameter Type	Symbol	Description
**Height parameters** only related to the statistical distribution of height values along the *z*-axis	*Sq*	Root mean square height of the surface
	Ssk	Skewness of height distribution
	Sku	Kurtosis of height distribution
	Sp	Maximum height of peaks
	Sv	Maximum height of valleys
	Sz	Maximum height of the surface
	Sa	Arithmetical mean height of the surface
**Spatial parameters** related to the directionality of data spatial periodicity	*Sal*	Fastest decay auto-correlation rate
	Str	Texture aspect ratio of the surface
**Hybrid parameters** related to the spatial organization of the data	*Sdq*	Root mean square gradient of the surface
	Sdr	Developed area ratio

2. Interaction terms in the experimental design-based factorial analysis

To study the effects of the three parameters used for the laser-modification of titanium surface on cell adhesion, a three-way ANOVA with full factorial design ($adhesion \sim distance * pattern * depth$) was carried out as a first explorative approach. Since the overall interaction term among the three factors (distance : pattern : depth) was not statistically significant (p-value = 0.883), the three-way ANOVA was re-run only considering the three pairwise interactions among factors. Even using such a model equation, no interaction term could be detected as statistically significant, though the coefficient of the product between pit depth and pattern type was very close to the usual, albeit conventional, 5% significance threshold (p-value = 0.052), suggesting a possible interaction between these two factors. In the absence of any (significant) evidence of interaction among the factors, a further three-way ANOVA with a plain additive model ($adhesion \sim distance + pattern + depth$) was used to study the main effects of the three independent variables. This analysis showed that all three parameters significantly affected cell adhesion, although to varying degrees. Complete summary statistics are reported in Table S2, along with the regression coefficients for the additive model, while the main effects of these three explanatory variables and the regression (hyper)plane are shown in Figure 5 of the main text.

Table S2. Statistics for the general linear model used to fit the data. The model is a purely additive three-way ANOVA since no significant interaction emerged from preliminary analyses.

Coefficients	Estimate	Std. Error	t-value	Adj. SS	F-value	p-value
**(Intercept)**	188.42	28.75	6.554	213005.04	42.949	$2.19 \cdot 10^{-06}$
**distance**	-69.42	28.75	-2.414	28912.04	5.830	$2.55 \cdot 10^{-02}$
**pattern**	65.75	28.75	2.287	25938.37	5.230	$3.32 \cdot 10^{-02}$
**depth**	111.08	28.75	3.864	74037.04	14.928	$9.67 \cdot 10^{-04}$

3. Model selection for the MLR based on roughness parameters

To be used as regressors in a multiple linear model of cell adhesion, the eleven roughness descriptors were first checked for multicollinearity (i.e., correlation among independent variables). Indeed, many Pearson correlation coefficients in the correlation matrix resulted very close to 1 (see Figure S1). This was rather expected, given the high degree of interdependence and redundancy in the definition of ISO 25178 descriptors. For example, $corr(Sq, Sa) = 1$ since both Sq and Sa represent an estimate of surface deviation from the mean pane, while $corr(Sz, Sp) = 0.93$ and $corr(Sz, Sv) = 0.95$ since $Sz = Sp + Sv$ by definition.

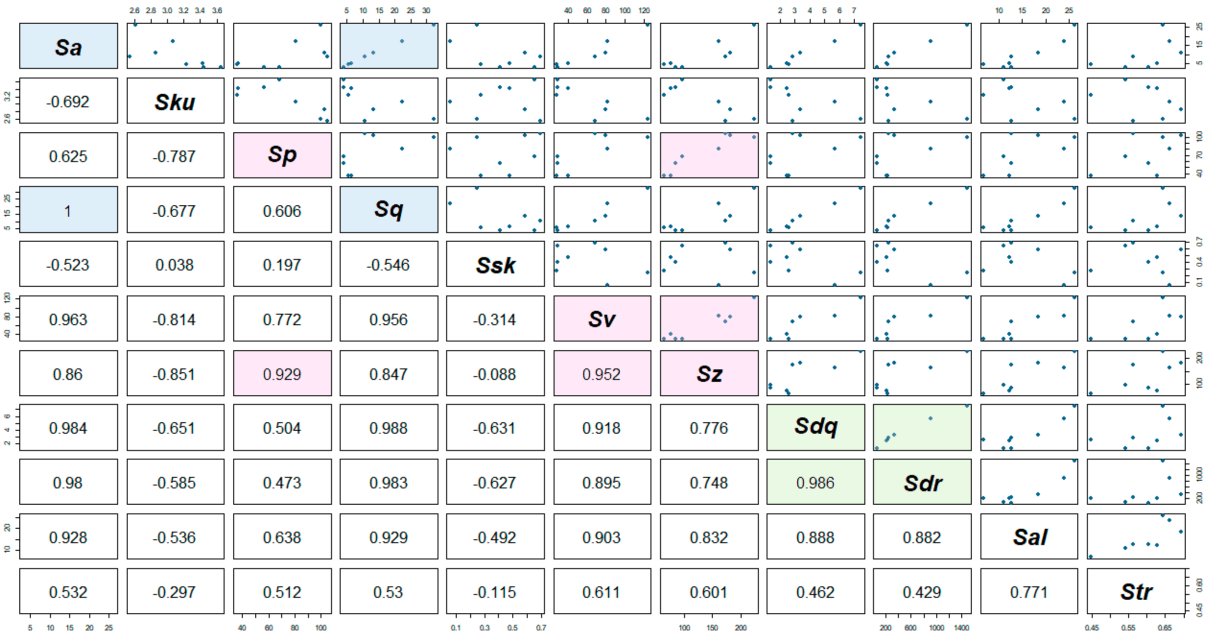

Figure S1. Correlation among regressors. Bottom-left panel: correlation matrix values (Pearson product moment). Upper-right panel: pairwise scatterplots for correlation detection. Diagonal elements: symbols of the eleven candidate ISO parameters to be used as statistical regressors. Background colors were used to highlight some examples of strong multicollinearity (i.e., high levels of correlation between independent variables).

Based on these considerations, the four most uninformative descriptors (Sa, Sdq, Sz, Sal) were removed from the dataset to make the number of retained feature one less than the number of samples (i.e., seven features evaluated for eight samples), an essential prerequisite for multiple linear regression (MLR). Thus, a first additive linear model was computed using all the seven retained explanatory variables (adhesion $\sim Sku + Sp + Sq + Ssk + Sv + Sdr + Str$) and the variance inflation factor (VIF) statistic for multicollinearity detection was used as a criterion for model selection. Specifically, we performed a multistep linear regression by progressively removing the regressors with the highest VIFs and stopping when $\text{VIF} < 4$ for all the predictors. This approach led to the removal of four more features (Sq, Sv, Sp, Sdr) leaving only three mutually unrelated descriptors to be used for the linear model: Sku, Ssk, and Str. Statistics of such multiple linear regression model are shown in Table S3.

Table S3. MLR summary statistics. Coefficient estimate, uncertainty, statistical significance, and VIF for each of the three uncorrelated ISO 25178 roughness descriptors used as regressors to predict cell adhesion.

Coefficients	Estimate	Std. Error	*t*-value	Adj. SS	*F*-value	*p*-value	*VIF*
**(Intercept)**	231.05	203.64	1.135	6427.06	1.287	$2.70\cdot10^{-01}$	

Sku	-94.68	39.94	-2.371	28062.15	5.621	$2.79·10^{-02}$	1.097
Ssk	-133.94	70.59	-1.898	17977.30	3.601	$7.23·10^{-02}$	1.013
Str	605.68	206.15	2.938	43100.15	8.632	$8.13·10^{-03}$	1.110
**Residuals**				99855.98			

4. High magnification of the protrusions surrounding the pits

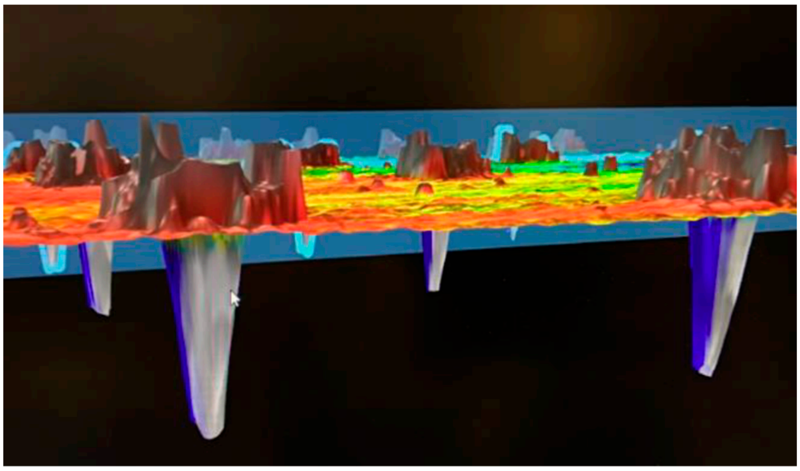

Figure S2. Edge peaks. High resolution 3D optical profilometry of a laser-patterned titanium surface for magnification of the protrusions (edge peaks) surrounding each pit.

5. Energy dispersive X-ray analysis (EDX)

Morphology of sample surfaces was investigated by scanning electron microscopy (SEM, Phenom XL G2 Desktop SEM, Thermo Fisher Scientific, Waltham, MA, USA). The microscope includes an EDX (energy dispersive X-ray analysis) tool that enabled the elemental analysis of the surface. The detector was configured in full BSD mode, the working distance was fixed at 8.43 mm, the accelerating voltage was set at 15 kV, and the chamber vacuum environment was maintained at 10 Pa.

EDX analysis was performed in triplicate on all titanium surfaces used in the study. The resulting percent composition values for carbon, titanium, and oxygen are shown in the bar chart of Figure S3.

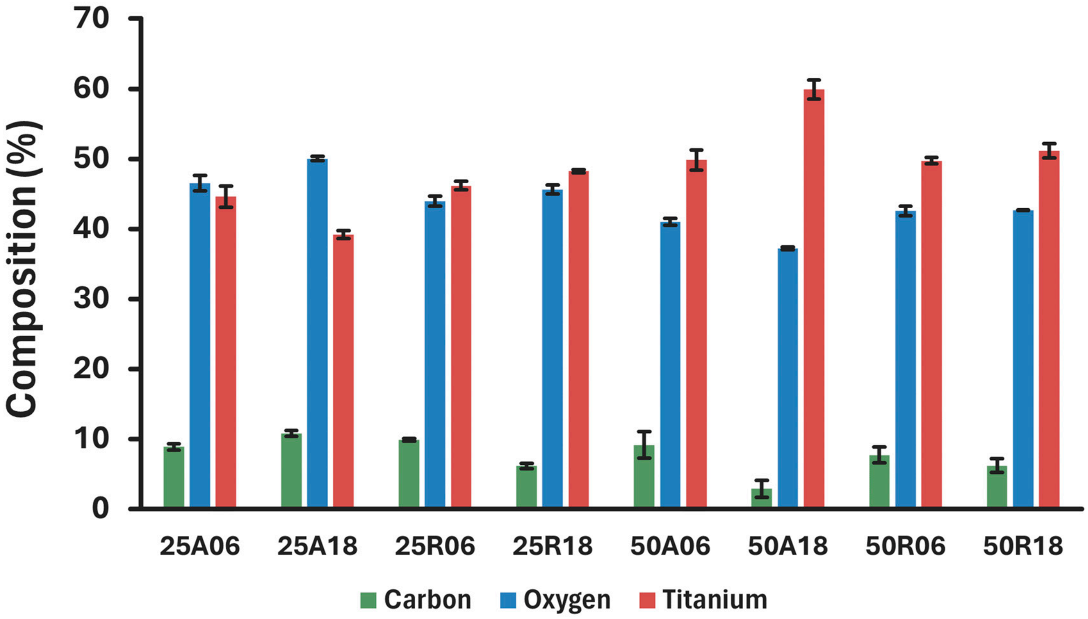

Figure S3. Elemental analysis of titanium disc surfaces. Percent composition values for carbon, oxygen, and titanium as assessed by energy dispersive X-ray analysis (EDX). Data from three independent measurements are represented as mean ± SE of percent presence of each element across the eight different titanium surfaces used in the study.

The presence of possible statistically significant differences in elemental composition between the different surfaces was established—for each element—through a series of pairwise *t*-tests, where the FWER (family-wise error rate) was controlled using the Bonferroni correction. Tables of all the adjusted *p*-values are shown below for each detected element (Table S4,5,6). Significant outcomes (*p*-values marked in red) were mostly related to the two surfaces with the highest and lowest oxygen presence, namely samples 25A18 and 50A18, respectively. No correlation emerged between the presence of Ti oxide on the surfaces and the cellular response assessed in terms of cell adhesion.

Table S4. Carbon relative presence. Bonferroni adjusted p-values resulting from the pairwise comparisons of the eight surface types with respect to the relative presence of carbon in the elemental composition (independent sample t-tests).

Carbon	25A06	25A18	25R06	25R18	50A06	50A18	50R06
25A18	0.137						
25R06	1.000	1.000					
25R18	0.038	0.004	0.020				
50A06	1.000	1.000	1.000	1.000			
50A18	1.000	1.000	1.000	1.000	0.576		
50R06	1.000	0.857	1.000	1.000	1.000	1.000	

**50R18**	0.837	0.229	0.573	1.000	1.000	1.000	1.000

Table S5. Oxygen relative presence. Bonferroni adjusted p-values resulting from the pairwise comparisons of the eight surface types with respect to the relative presence of oxygen in the elemental composition (independent sample t-tests).

Oxygen	25A06	25A18	25R06	25R18	50A06	50A18	50R06
**25A18**	0.653						
**25R06**	0.913	0.035					
**25R18**	1.000	0.052	1.000				
**50A06**	0.148	0.001	0.159	0.021			
**50A18**	0.091	0.000	0.047	0.018	0.050		
**50R06**	0.263	0.016	1.000	0.126	1.000	0.070	
**50R18**	0.693	0.015	1.000	0.404	0.785	0.393	1.000

Table S6. Titanium relative presence. Bonferroni adjusted p-values resulting from the pairwise comparisons of the eight surface types with respect to the relative presence of titanium in the elemental composition (independent sample t-tests).

Titanium	25A06	25A18	25R06	25R18	50A06	50A18	50R06
**25A18**	0.431						
**25R06**	1.000	0.004					
**25R18**	1.000	0.009	0.540				
**50A06**	0.342	0.064	0.898	1.000			
**50A18**	0.091	0.465	0.709	1.000	0.274		
**50R06**	0.566	0.001	0.053	0.472	1.000	1.000	
**50R18**	0.147	0.008	0.105	0.915	1.000	0.683	1.000
